# Dose-extending placebo effect in a rat model of buprenorphine maintenance treatment

**DOI:** 10.1007/s00213-025-06815-w

**Published:** 2025-05-21

**Authors:** Kayla M. Pitts, Emma M. Pilz, Luana Colloca, Yavin Shaham, Jonathan J. Chow

**Affiliations:** 1 Behavioral Neuroscience Branch, Intramural Research Program, NIDA, NIH, Baltimore, MD USA; 2 Department of Neuroscience, Feinberg School of Medicine, Northwestern University, Chicago, IL USA; 3 School of Nursing, University of Maryland, Baltimore, MD USA

**Keywords:** Drug self-administration, Placebo, Opioid maintenance treatment, Buprenorphine, Remifentanil

## Abstract

**Rationale and Objective:**

Clinical studies have shown that exposure to placebos or combining placebos with a lower medication dose can mimic the effect of a higher effective medication dose. This "dose-extending placebo effect" has been demonstrated in treatment for pain and other medical conditions but not in addiction. Here, we tested if a "dose-extending placebo effect" occurs in a rat model of opioid (buprenorphine) maintenance.

**Methods:**

We trained 27 rats to self-administer remifentanil (5 µg/kg/infusion, 1-h per day). Next, we implanted some rats with buprenorphine minipumps (3 mg/kg/day, Exp. 1) or pretreated others with daily intravenous buprenorphine (0.3 mg/kg, Exp. 2), and introduced a discriminative cue (houselight + tone) during the self-administration sessions (the buprenorphine-maintenance cue). After discontinuing buprenorphine treatment, we retrained the rats for remifentanil self-administration without the cue. Next, we tested the effect of low and high buprenorphine doses (0.15 and 0.3 mg/kg), the buprenorphine-maintenance cue, and the combination of the low-dose with the cue on remifentanil self-administration.

**Results:**

Rats learned to self-administer remifentanil, and buprenorphine maintenance suppressed drug self-administration. The low buprenorphine dose modestly decreased self-administration, while the high dose caused a strong inhibition. Tests for the "dose-extending placebo effect" showed that discriminative buprenorphine cue alone had no effect, while the low dose plus the buprenorphine cue mimicked the inhibitory effect of the high dose.

**Conclusions:**

This proof-of-concept study suggests that a "dose-extending placebo effect" can be modeled in rats undergoing opioid maintenance. This approach could support dose-reduction strategies in humans undergoing opioid maintenance therapy.

## Introduction

Placebo effects across various medical conditions strongly influence medical outcomes (Colloca 2019). For example, individuals who believe in the effectiveness of a pain relief treatment often report pain reduction, regardless of whether they receive an active drug or a placebo (Benedetti et al. 2007; Price et al. 1999). Similar placebo-like effects have also been observed in rat models (Herrnstein 1962; Keller et al. 2018). For example, after repeated pairings of morphine with taste or odor cues, or cyclosporine with a taste cue, exposure to these cues can induce morphine-like analgesic effects (Bardo and Valone 1994; Valone et al. 1998) or cyclosporine-like immunosuppressive effects (Pacheco-Lóipez et al. 2009).

One emerging therapeutic application of the placebo effect is the “dose-extending placebo” approach. This method involves using stimuli—such as treatment settings, positive verbal reinforcement from physicians, or placebo pills—that become associated with treatment outcomes through repeated pairings with an active medication (Colloca et al. 2016). These stimuli serve as “dose extenders,” prolonging the treatment’s effectiveness while reducing the overall medication dosage. In studies using this approach, participants receive full medication doses on some treatment days and either a placebo alone (Ader et al. 2010; Morales-Quezada et al. 2020; Perlis et al. 2015) or a placebo combined with a lower medication dose on other days (Sandler and Bodfish 2008). Variations of this approach using placebos have demonstrated efficacy in combination with corticosteroids for psoriasis (Ader et al. 2010), zolpidem for insomnia (Perlis et al. 2015), stimulants for attention deficit hyperactivity disorder (ADHD) (Sandler and Bodfish 2008), and morphine for surgical pain induced by spinal cord injury (Morales-Quezada et al. 2020).

Recently, a placebo-based approach was explored in opioid addiction treatment. In this study, researchers used an open-label placebo procedure to determine if adding a placebo to standard methadone treatment would reduce overall methadone intake (Belcher et al. 2023). In open-label placebo studies, participants are explicitly informed that they are receiving a placebo (Colloca 2019). Belcher et al. (2023) reported that while the open-label placebo had no effect on the primary outcome (overall methadone intake), it did improve secondary outcomes such as treatment retention and self-reported sleep quality. However, a formal dose-extending procedure—where a placebo substitutes the medication on certain days or is combined with a lower dose—has not yet been tested in drug addiction treatment.

To our knowledge, no published studies have investigated the placebo effect as an adjunct to pharmacological treatments in rat models of drug addiction. In this proof-of-concept study, we tested if two variations of the dose-extending procedure (placebo substitution and placebo combined with a low-dose medication) would mimic the inhibitory effects of chronic buprenorphine on remifentanil self-administration in a rat model of opioid maintenance (Bossert et al. 2020; Sorge et al. 2005).

## Materials & Methods

### Subjects

In Exp. 1, we used 15 (7 female) Sprague–Dawley rats, and in Exp. 2, we used 12 (6 female) Sprague–Dawley rats (estimated age at arrival: ~ 7–8 weeks PND; body weight at arrival: males, 201–225 g; females, 176–200 g; Charles River). The rats arrived pair-housed and acclimated together for one week, after which we separated and single-housed the rats. Approximately one week before the start of the experiments, we handled the rats daily. We performed all experiments in accordance with the NIH Guide for the Care and Use of Laboratory (8 th edition), under protocols approved by the NIDA IRP Animal Care and Use Committee.

### Apparatus

The experiments were conducted in operant chambers (Med Associates) controlled by Med-PC IV. We outfitted the front panel of the operant chamber with a recessed pellet receptacle (ENV-200R1M) equipped with a head entry detector (ENV-254-CB) and two retractable levers (ENV-112 CM) mounted on either side. A mounted dispenser (ENV-203 M-45) delivered food pellets. Above each lever was a white cue-light (ENV-221 M) and above the recessed pellet receptacle, at the top of the panel, a white houselight, though this was not used in the current experiment. At the very top right corner of the front panel (above the right white cue-light and lever) was a tone generator (ENV-223 AM; 2.9 kHz). A red houselight (ENV-221RD) was placed in the middle-top of the back panel. Each operant chamber had a syringe pump (PHM-200) that was located on top of the operant chambers and connected to a custom made swiveled-tether (PHM-115IP).

### Drugs

We received remifentanil hydrochloride and buprenorphine hydrochloride from the NIDA-IRP pharmacy. We dissolved remifentanil in sterile saline. In Exp. 1 (minipumps), we dissolved buprenorphine in sterile water. In Exp. 2 (daily injections), we dissolved buprenorphine in sterile saline. We based the doses of remifentanil and buprenorphine on our previous studies (Bossert et al. 2020; Chow et al. 2022; Chow and Beckmann 2021).

## General procedures

### Intravenous catheterization surgery

We first injected rats with ketoprofen (2.5 mg/kg, s.c.; Covetrus) ~ 1 h before surgery. We then anesthetized the rats with isoflurane gas (5% induction, 2–3% maintenance; Covetrus). We then inserted silastic catheters into the right jugular vein and secured the catheter to the vein with a sterile suture. The catheter was passed subcutaneously through the right shoulder and out the back where it was connected to a custom-modified 22-gauge catheter port. After completion of the surgery, we injected the rats with ketoprofen (2.5 mg/kg, s.c.) and 3 ml sterile saline. We then conducted postoperative care which lasted for 1 week consisting of 3 consecutive days of ketoprofen (2.5 mg/kg, s.c.) and 6 consecutive days intravenous infusions of 0.2 ml gentamicin (5 mg/ml dissolved in sterile saline; Fresenius Kabi) via the catheter port.

### Remifentanil self-administration training (Exp. 1–2)

**Fig. 1:**
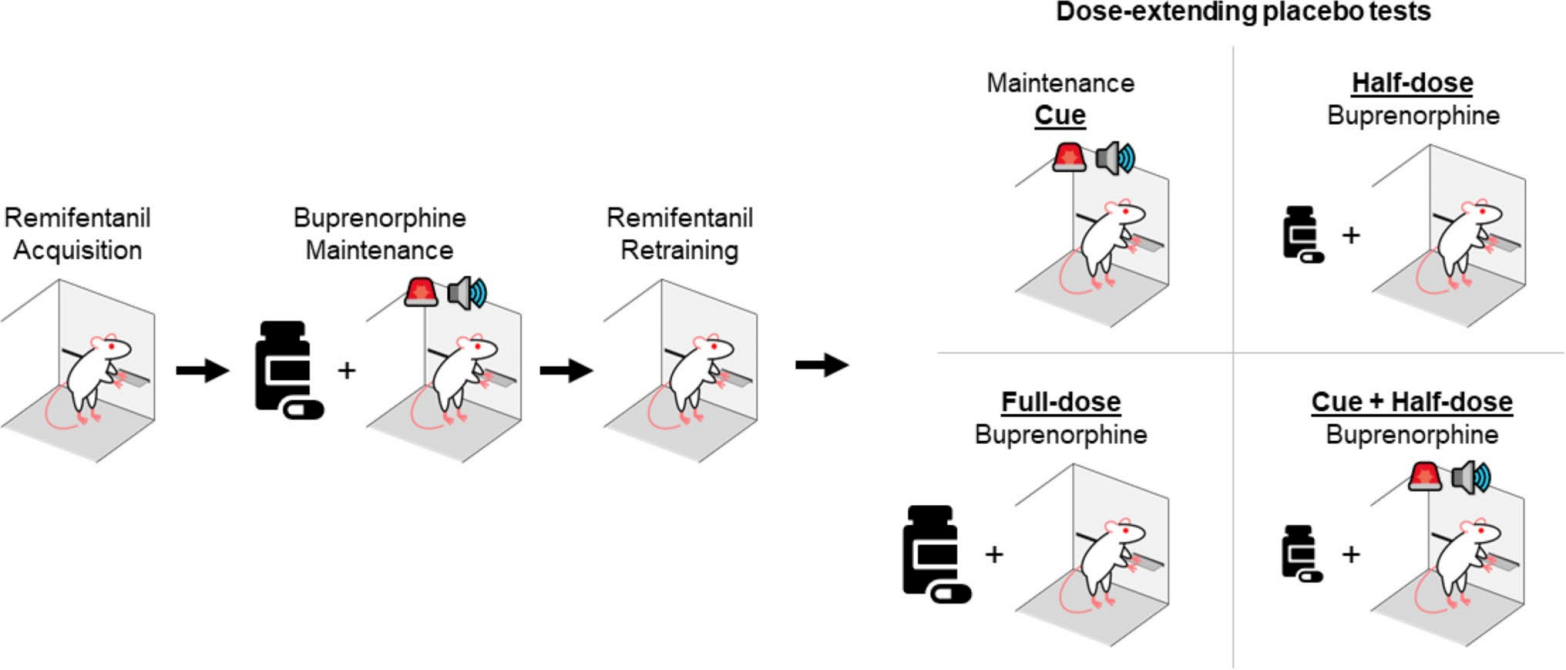
Schematic of the experimental design. We trained rats to self-administer remifentanil, then placed them on buprenorphine maintenance treatment (osmotic minipumps or daily intravenous injections) while continuing to train the rats to self-administer remifentanil but in the presence of a cue (red houselight and tone, buprenorphine maintenance cue). After maintenance, we retrained the rats for remifentanil self-administration and then tested them under the following conditions: in the presence of the buprenorphine maintenance Cue, after Half-dose or Full-dose buprenorphine pretreatment, and with both the maintenance cue and the Half-dose pretreatment (Cue + Half-dose)

**Fig. 2:**
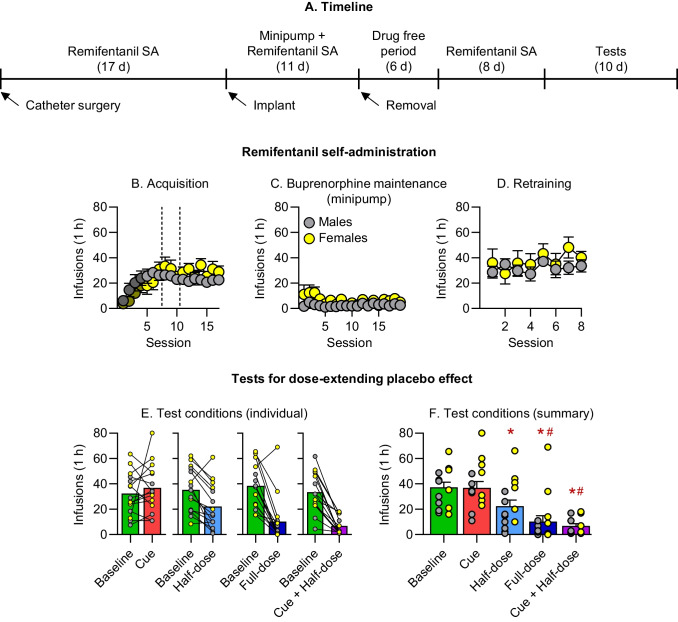
Experiment 1. **A.** Timeline of Experiment 1. Remifentanil self-administration during **B.** acquisition. Dotted line represents FR change (1→3→5) and darker symbols indicate 10 µg/kg/infusion remifentanil. **C.** buprenorphine maintenance via osmotic minipumps, **D.** retraining following minipump removal and a drug free break; data are Mean ± SEM. **E**. Individual remifentanil self-administration in males (gray) and females (yellow) during baseline (2-session average prior to each individual test) and tests. **F.** Remifentanil self-administration during Baseline (average of the four baselines prior to tests) and the test conditions; data are Mean ± SEM. * Different from Baseline and Cue, p < 0.05; ^#^ Different from Half-Dose, p < 0.05

**Fig. 3:**
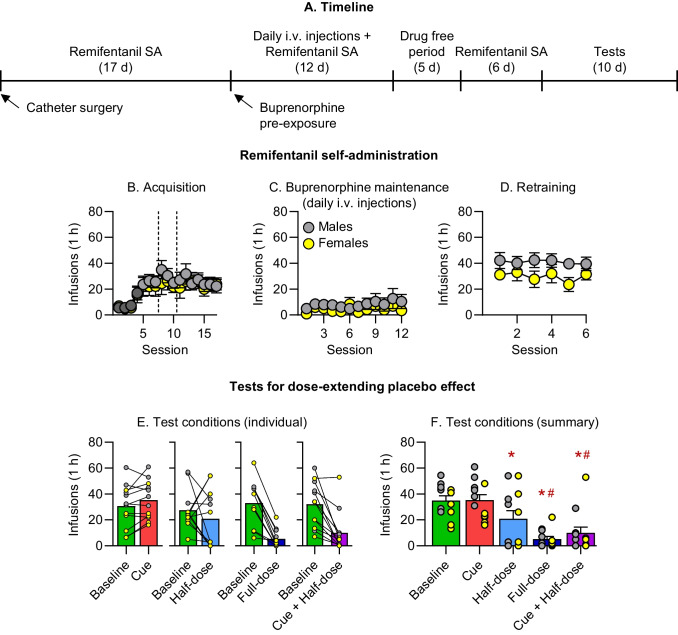
Experiment 2. **A.** Timeline of Experiment 2. Remifentanil self-administration during **B.** acquisition. Dotted line represents FR change (1→3→5) and darker symbols indicate 10 µg/kg/infusion remifentanil. **C.** buprenorphine maintenance via daily i.v. pretreatments, **D.** retraining following cessation of pretreatments and a drug free break; data are Mean ± SEM. **E**. Individual remifentanil self-administration in males (gray) and females (yellow) during baseline (2-session average prior to each individual test) and tests. **F.** Remifentanil self-administration during Baseline (average of the four baselines prior to tests) and the test conditions; data are Mean ± SEM. * Different from Baseline and Cue, p < 0.05 or p = 0.075 (Half-dose vs. Baseline); ^#^ Different from Half-Dose, p < 0.05

After postoperative care, we trained the rats for remifentanil self-administration on a fixed ratio (FR) schedule of reinforcement. All rats were intravenously flushed with 0.1 ml sterile saline via catheter port prior to being tethered to the drug self-administration line and placed into the operant chambers. Each session began with the extension of a single lever (counterbalanced across subjects). During each trial, completion of the FR requirement resulted in the retraction of the lever, delivery of 0.1 ml remifentanil, and the illumination of the cue light located above the drug-paired lever for 5.5 s which matched the infusion time. Each trial was separated by a 15-s intertrial interval (ITI), which includes the duration of the infusion and cue light illumination. We trained the rats on an FR1 schedule for 10 µg/kg/infusion remifentanil for 4 daily sessions, then continued at an FR1 schedule but for 5 µg/kg/infusion remifentanil for 3 daily sessions, then at FR3 schedule for 5 µg/kg/infusion remifentanil for another 3 daily sessions, and then finally at a FR5 for 5 µg/kg/infusion remifentanil for 7 daily sessions. After each 1-h session, we flushed the rats’ catheters with 0.2 ml gentamicin prior to being returned to their home cages. We selected remifentanil because it is a fast-acting opioid agonist (Haidar et al. 1997) that is reliably self-administered at high rates by rats and monkeys (Ko et al. 2002; Panlilio and Schindler 2000) and induces rapid opioid-related subjective effects in humans (Baylon et al. 2000; Lile et al. 2024). For a general overview of the phases of training and testing see Fig. 1. For a general breakdown of the days of the experiments see Figs. 2A and 3A.

### Experiment 1: Buprenorphine treatment via minipumps

#### Remifentanil self-administration under buprenorphine treatment (minipumps)

The next day, after the last daily session of remifentanil self-administration training, we implanted the rats with osmotic minipumps (Model 2ML2, Alzet) filled with buprenorphine (3 mg/kg/d which is 0.125 mg/kg/h). We anesthetized the rats with isoflurane gas (5% induction, 2–3% maintenance) and conducted minipump surgery as previously described (Chow et al. 2024). Briefly, we made a small incision on the lower right side. We used a hemostat to spread apart the subcutaneous connective tissue to make a small pocket for the pump. We placed the osmotic pumps into the pocket with the flow moderator directed away from the incision and then closed the incision with sterile wound clips. We then injected the rats with ketoprofen (2.5 mg/kg, s.c.).

The day after minipump implantation, we trained the rats for remifentanil self-administration. Each session now began with the onset of the buprenorphine treatment cue, which consisted of a red houselight and 29 kHz tone that were on continuously throughout the entire 1-h sessions. Each session followed the same procedure as in the initial training: completion of the response requirement caused the lever to retract, delivered 0.1 ml of remifentanil over 5.5 s, and illuminated the cue-light, followed by a 15-s ITI. However, during this phase, we trained the rats on an FR10 schedule with 5 µg/kg/infusion remifentanil for 19 sessions. We increased the FR requirement from 5 to 10 to increase the discriminability of the maintenance phase, such that the interoceptive effects of buprenorphine and decreased rate of reinforcement (Kearns et al. 2005) would aid in the inhibitory association of the maintenance cue. The first 3 sessions were conducted on separate days, while the remaining 16 sessions were conducted over 8 days twice daily with morning (~ 9 AM) and afternoon (~ 1 pm) sessions. We kept the rats in their homecage during the period between the two daily sessions.

#### Remifentanil retraining and assessment of a “dose-extending” placebo effect

The day after the last session of remifentanil self-administration under treatment conditions (i.e., red houselight and tone at FR10 schedule for 5 µg/kg/infusion remifentanil), we anesthetized the rats with isoflurane gas (5% induction, 2–3% maintenance) and removed the minipumps. Afterward, we gave the rats a 6-d drug-free break before retraining them for remifentanil self-administration under the initial conditions (i.e., single-lever extension for 5 µg/kg/infusion remifentanil at FR5 schedule); during the break, we flushed the rats daily with 0.2 ml gentamicin. We retrained rats for 8 sessions over 6 days (2 daily sessions followed by another 3 twice-daily sessions [~ 9 AM and ~ 1 PM] as described above)—this was done to ensure all training and subsequent testing could be completed prior to any concerns regarding catheter patency.

After retraining, we tested the rats under four different conditions: (1) the buprenorphine maintenance cue (Cue), (2) ~ 50% of the calculated hourly dose of buprenorphine during maintenance (Half-dose), (3) ~ 100% of the calculated hourly dose of buprenorphine during maintenance (Full-dose), and (4) the combination of the maintenance cue and the Half-dose (Cue + Half-dose). Remifentanil self-administration during these tests was exactly like training and retraining. Each 1-h session began with the extension of the same previously trained remifentanil-associated lever, and completion of the FR5 schedule resulted in the retraction of the lever, delivery of 5 µg/kg/infusion remifentanil, and the illumination of the cue light located above the drug-paired lever for 5.5 s; each trial was separated by a 15-s ITI.

For the Cue test, we flushed the rats’ catheters with 0.1 ml sterile saline ~ 3.5 h before the start of the session and presented the red houselight and tone (Cue) during the entire 1-h session. For the Half- and Full-dose tests, we injected the rats intravenously with 0.125 mg/kg or 0.3 mg/kg buprenorphine (0.1 ml), respectively, ~ 3.5 h before the start of the session followed by 0.1 ml sterile saline. For the Cue + Half-dose test, we injected the rats intravenously with 0.125 mg/kg buprenorphine (0.1 ml) ~ 3.5 h before the start of the session followed by 0.1 ml sterile saline and presented the Cue during the entire session.

We chose the pretreatment time based on the half-life of buprenorphine in rats (Ohtani 2007) with an estimated buprenorphine level of 0.0625 mg/kg (Half-dose) or 0.125 mg/kg (Full-dose) at the start of the session. The ‘Full-dose’ approximates the hourly buprenorphine delivery via the minipump (0.125 mg/kg/h), while the ‘Half-dose’ approximates half the hourly buprenorphine delivery via the minipump (0.0625 mg/kg).

We conducted the test conditions on four separate days using a pseudo-Latin square design where the order of treatments was repeated in some rats. Between the test days, the rats continued remifentanil self-administration (FR5 schedule for 5 µg/kg/infusion) for 4 sessions over 2 days (~ 9 AM and ~ 1 PM).

### Experiment 2: Buprenorphine treatment via daily i.v. pretreatments

#### Remifentanil self-administration under buprenorphine treatment (daily i.v. pretreatments)

In Exp. 1, we found evidence of a dose-extending placebo effect using continuous subcutaneous (s.c.) buprenorphine delivery via a minipump as the opioid maintenance condition. In Exp. 2, we determined if this effect would also occur when the buprenorphine maintenance cue was repeatedly paired with a different maintenance-like condition—daily repeated systemic buprenorphine injections. Based on the results of Exp. 1, we selected the 0.3 mg/kg as the daily maintenance dose of buprenorphine. The day after the last session of remifentanil self-administration training, we injected the rats i.v. via catheter ports with 0.1 ml of 0.3 mg/kg buprenorphine followed by 0.1 ml sterile saline and returned them to their homecages. The day after i.v. buprenorphine exposure, we trained the rats for remifentanil self-administration in the same treatment context as described above (continuous red houselight and tone at FR10 for 5 µg/kg/infusion remifentanil for 1 h). However, ~ 3.5 h before the start of these sessions (Ohtani 2007), we injected the rats intravenously with 0.3 mg/kg buprenorphine (0.1 ml) followed by 0.1 ml sterile saline via the catheter port to achieve ~ 0.125 mg/kg buprenorphine level at the start of the session to approximate Exp. 1. We trained the rats under this treatment regimen for 12 daily sessions.

#### Remifentanil retraining and assessment of a “dose-extending placebo” effect

After the last session of remifentanil self-administration under the treatment conditions (i.e., red houselight and tone at FR10 schedule for 5 µg/kg/infusion remifentanil), we gave the rats a 5-d drug-free break before retraining them for remifentanil self-administration under the initial conditions (i.e., single-lever extension for 5 µg/kg/infusion remifentanil at FR5 schedule); during the break, we flushed the rats’ catheters daily with 0.2 ml gentamicin. We then retrained the rats for 6 sessions over four days (2 daily sessions followed by another 2 twice-daily sessions [~ 10 AM and ~ 2 PM] as described above).

Finally, we tested the rats under the Cue, Half-dose, Full-dose, and Cue + Half-dose conditions with continued twice-daily remifentanil self-administration sessions between tests as described in Exp. 1.

### Data analysis

We analyzed the test data with repeated-measures ANOVAs using SPSS (Version 30, GLM procedure). We followed significant main effects (p < 0.05) with Fisher’s protected least significant differences (PLSD) test. After obtaining a significant effect in the overall repeated-measures ANOVA, we used Fisher PLSD to test two a priori hypotheses regarding the dose-extending placebo effect. The first hypothesis is that the Cue (placebo) will mimic the effect of the half-dose on remifentanil self-administration. The second is that the Cue + Half-dose (placebo combined with a low-dose) will mimic the effect of the high-dose on remifentanil self-administration.

We used both male and female rats in Exp. 1 and 2. For transparency, we show data for both sexes in the figures and included Sex as a between-subject variable in the statistical analyses. For complete statistical reporting, see Table 1.

**Table 1:** Statistical analysis of the data presented in Figs. 2–3

Figure Number	Factor Name	F-value	*p*-value
Figure 2B. Exp. 1: Acquisition	We did not perform a formal statistical analysis because we changed both the unit dose and FR requirements during training (see Methods)		
Figure 2C. Exp.1: Maintenance	Sex (male, female), between-subject Session (1–19), within-subject Sex x Session	F_(1,13)_ = 7.43 F_(18,252)_ = 1.66 F_(18,234)_ = 0.98	0.017* 0.05* 0.49
Figure 2D. Exp.1: Retraining	Sex (male, female), between-subject Session (1–8), within-subject Sex x Session	F_(1,13)_ = 0.54 F_(7,98)_ = 1.61 F_(7,91)_ = 1.19	0.48 0.14 0.32
Figure 2E. Exp. 1. Test: Baseline responding	Sex (male, female), between-subject Baseline before Cue, Half dose, Full dose, and Cue + Half dose, within-subject Sex x Baseline	F_(1,13)_ = 0.02 F_(3,42)_ = 1.13 F_(3,39)_ = 0.80	0.97 0.35 0.50
Figure 2F. Exp. 1: Test	Sex (male, female), between-subject Test condition Baseline, Cue, Half dose, Full dose, Cue + Half dose), within-subject Test x Sex	F_(1,13)_ = 1.88 F_(4,56)_ = 23.17 F_(4,52)_ = 1.36	0.19 < 0.001* 0.26
Figure 3B. Exp. 2: Acquisition	We did not perform a formal statistical analysis because we changed both the unit dose and FR requirements during training (see Methods)		
Figure 3C. Exp.2: Maintenance	Sex (male, female), between-subject Session (1–12), within-subject Sex x Session	F_(1,10)_ = 0.89 F_(11,121)_ = 1.09 F_(11,110)_ = 0.48	0.37 0.37 0.91
Figure 3D. Exp. 2: Retraining	Sex (male, female), between-subject Session (1–6), within-subject Sex x Session	F_(1,10)_ = 2.83 F_(5,55)_ = 0.87 F_(5,50)_ = 0.68	0.12 0.51 0.64
Figure 3E. Exp. 2: Test: Baseline responding	Sex (male, female), between-subject Baseline before Cue, Half dose, Full dose, and Cue + Half dose, within-subject Sex x Baseline	F_(1,10)_ = 4.32 F_(3,33)_ = 0.80 F_(3,30)_ = 1.62	0.064 0.50 0.21
Figure 3F. Exp. 1: Test	Sex (male, female), between-subject Test condition (Baseline, Cue, Half dose, Full dose, Cue + Half dose), within-subject Test x Sex	F_(1,10)_ = 0.94 F_(4,44)_ = 16.30 F_(4,40)_ = 2.12	0.36 < 0.001* 0.10

## Results

### Acquisition, maintenance treatment, and retraining

In both experiments, the rats learned to lever press for remifentanil infusions (Figs. 2B and 3B). Remifentanil self-administration was strongly suppressed by chronic delivery of buprenorphine via minipumps or daily intravenous injections (Figs. 2C and 3C). During retraining, remifentanil self-administration resumed to levels similar or slightly higher than the acquisition phase (Figs. 2D and 3D). We observed sex differences during the maintenance phase of Exp. 1 [F(1,13) = 7.4, *p* = 0.02] with females less sensitive to the suppressive effect of buprenorphine than males. No significant sex differences were observed for acquisition and retraining in Exp. 1 and 2, and maintenance in Exp. 2.

These results indicate that rats learned to self-administer remifentanil, significantly decreased remifentanil self-administration during buprenorphine maintenance treatment, and then maintained remifentanil self-administration after the cessation of buprenorphine treatment.

### Assessment of a “dose-extending placebo” effect

In both Exp. 1 and Exp. 2, we first assessed if there were any changes across baselines prior to each test (Figs. 2E and 3E). Baseline was defined as the mean number of infusions during the two sessions prior to each test, and we analyzed the baselines prior to Cue, Half-dose, Full-dose, Cue + Half-dose tests as a within-subjects factor and Sex as a between-subjects factor. The analyses showed no significant differences in Baseline or interaction between Baseline and Sex (*p values* > 0.05), indicating that remifentanil self-administration remained stable prior to each test in both experiments. However, baseline self-administration in males was somewhat higher than in females in Exp. 2 [main effect of Sex, F(1,10) = 4.3, *p* = 0.064] but not Exp. 1 [F(1,13) = 0.02, *p* = 0.97].

To assess if a “dose-extending placebo” effect was observed (Figs. 2F and 3F), we analyzed the data using Test Condition (Baseline, Cue, Half-dose, Full-dose, Cue + Half-dose) as a within-subjects factor and Sex as the between-subjects factor. Baseline for this analysis refers to the mean of the baselines (2 sessions) prior to each test condition. The analysis showed a significant effect of Condition for both Exp. 1 [F(4,56) = 23.2, *p* < 0.001] and Exp. 2 [F(4,44) = 16.3, *p* < 0.001]. There were no significant main effects of Sex or Sex x Test condition interaction (*p values* > 0.05).

For Exp. 1 and 2, post-hoc analyses across sexes showed that there were no significant differences between Baseline and Cue test conditions [*p values* > 0.05]. In Exp. 1, the response in the Half-dose condition was significantly lower than at Baseline, while in Exp. 2, this difference was only marginally significant [t(14) = 4.3, *p* < 0.001 and t(11) = 2.0, *p* = 0.075]. The Half-dose was lower than the Cue [t(14) = 4.5, *p* < 0.001 and t(11) = 2.3, *p* = 0.042] condition, and higher than both the Full-dose [t(14) = 2.9, *p* = 0.012 and t(11) = 3.1, *p* = 0.009] and Cue + Half-dose [t(14) = 3.6, *p* = 0.003 and t(11) = 2.5, *p* = 0.031] conditions. Reported values are in reference to Exp. 1 and Exp. 2, respectively. In both experiments, there were no significant differences between the Full-dose and the Half-dose + Cue [*p values* > 0.05].

Overall, these findings indicate that the buprenorphine-associated maintenance cue (red houselight and tone) alone had no effect on remifentanil self-administration and thus did not show a placebo-like effect. In contrast, combining the maintenance cue with the Half-dose of buprenorphine, mimicked the suppressive effect of the Full-dose condition. This effect was somewhat stronger in rats previously exposed to buprenorphine via minipumps compared to those receiving repeated daily buprenorphine injections.

## Discussion

We examined two potential forms of the "dose-extending placebo" effect in a rat model of buprenorphine maintenance (Bossert et al. 2020). Specifically, we tested if a discriminative cue (red houselight and tone) previously associated with a buprenorphine dose (Full-dose) that strongly suppresses remifentanil self-administration would (1) suppress self-administration on its own and (2) enhance the effect of a lower buprenorphine dose (Half-dose).

We hypothesized that the placebo (the buprenorphine maintenance cue) would moderately suppress remifentanil self-administration, while the placebo paired with a lower dose would strongly suppress self-administration, mimicking the effect of the full dose. Our results partially confirmed this hypothesis: the maintenance cue alone had no effect on remifentanil self-administration, but when paired with the lower buprenorphine dose, it produced suppression comparable to the full dose. This dose-extending placebo effect was observed across two different administration routes (osmotic minipumps and daily buprenorphine injections), demonstrating its generality.

We found little evidence for sex differences in dose-extended placebo under our experimental conditions, consistent with human studies reporting minimal sex differences in placebo responses (Enck and Klosterhalfen 2019). Similarly, we did not observe sex differences in remifentanil self-administration, which agrees with our previous findings with other opioid drugs, including heroin (Bossert et al. 2024, 2023), oxycodone (Fredriksson et al. 2020, 2023), and fentanyl (Claypool et al. 2023; Reiner et al. 2021). However, we found that females were less sensitive than males to the suppressive effects of buprenorphine on remifentanil self-administration when delivered via osmotic minipumps (Fig. 2B) but not through repeated intravenous injections (Fig. 3B). The reasons for this sex-specific effect based on the route of administration are unknown.

### Mechanisms of the dose-extending placebo effect

We initially hypothesized that the buprenorphine-associated cue would act as a conditioned inhibitor—a stimulus predicting the absence of an expected outcome (Rescorla 1969)—thereby suppressing remifentanil self-administration. Prior work by Kearns et al. (2005) demonstrated that discriminative cues signaling the absence of cocaine reinforcement can serve as conditioned inhibitors, reducing cocaine seeking in rats. However, our findings do not support this hypothesis. In our study, the maintenance cue signaled the presence of buprenorphine, which reduces the reinforcing effect of opioids (e.g., remifentanil), and increased FR requirement to indicate a context where remifentanil self-administration is not rewarding or less rewarding. Yet, when presented on its own, the cue failed to suppress remifentanil self-administration, indicating that it did not function as a conditioned inhibitor. A key procedural difference is that the rats in Kearns et al. (2005) study were trained with a discriminative cue that explicitly signaled the unavailability of cocaine. In contrast, in our experiment, the rats could continue to self-administer remifentanil during buprenorphine treatment.

When the maintenance cue was paired with a lower buprenorphine dose (Cue + Half-dose condition), a dose-extending placebo effect emerged, leading to suppression levels like the Full-dose condition. Two potential learning mechanisms may explain this effect. The first is configural learning (Pearce 1987) which suggests that when the maintenance cue is paired with buprenorphine’s interoceptive effects, an integrated representation is formed, functioning as a new stimulus rather than separate elements. The fact that the cue alone had no effect suggests that its influence depends on the interoceptive state induced by buprenorphine. Drugs can act as interoceptive stimuli (Bouton and Swartzentruber 1991; Overton 1974; Stolerman 1992), and the low buprenorphine dose may generalize to the full dose in the presence of the cue, creating a similar interoceptive state (Thompson et al. 2019). However, since the low-dose alone had a moderate suppressive effect (Half-dose condition), it remains unclear whether configural learning accounts for our results.

Another explanation is occasion setting (Holland 1992). Occasion setters are stimuli that do not directly elicit behavior but instead determine when other stimulus-outcome or response-outcome relationships are in effect. In our study, the maintenance cue did not directly suppress remifentanil self-administration but enhanced the suppressive effect of the lower buprenorphine dose, leading to suppression comparable to the Full-dose condition. This suggests that the maintenance cue functioned as an occasion setter, modulating buprenorphine’s suppressive effect.

Finally, the neuropharmacological mechanisms underlying the dose-extending placebo effect observed in our operant model are unknown. We speculate that the activation of endogenous opioid systems—previously implicated in pain- and morphine-related placebo effects in both humans and animal models (Colloca 2019; Keller et al. 2018)—plays a critical role. In agreement with this idea, Nolan et al. (2012) reported a naloxone-dependent, placebo-like analgesic effect of systemic saline injections following repeated morphine administration in an operant model where access to a sweetened milk reinforcer was contingent on facial contact with a heated thermode. We also speculate that dopamine likely plays a role in the dose-extended placebo effect. Pharmacological studies have reported that dopamine agonists and antagonists have minimal effects on placebo-related analgesia in humans (Kunkel et al. 2024; Zunhammer et al. 2018). However, dopamine activity is critical to placebo effects observed in Parkinson’s treatment with apomorphine (Benedetti et al. 2004; de la Fuente-Fernandez et al. 2001). Additionally, many studies have demonstrated a role of dopamine in both classical and operant conditioning responses to opioid drugs (Badiani et al. 2011; Di Chiara 1999; Reiner et al. 2019; Stewart 1992).

### Methodological and translational considerations

One methodological consideration is that, during the maintenance phase, the discriminative cue was associated with both buprenorphine exposure and a higher response requirement (FR10). However, we believe that the increased FR requirement had minimal influence on the test results, because the Full-dose condition strongly suppressed remifentanil self-administration during testing under an FR5 schedule, to levels comparable to those seen during maintenance under the FR10 schedule (Fig. 2C vs. 2 F, and Fig. 3C vs. 3 F). Another methodological limitation is the absence of a buprenorphine vehicle condition that was paired with the discriminative cue during the maintenance phase. However, it is unlikely that a cue associated with a vehicle alone would affect remifentanil self-administration during testing, because the cue paired with buprenorphine was ineffective when presented on its own (Figs. 2E-F and 3E-F). Additionally, previous placebo studies in rats have shown that unconditioned cues do not induce opioid-like responses (Bardo and Valone 1994; Valone et al. 1998).

From a translational perspective, our findings suggest that incorporating dose-extending placebo strategies into opioid maintenance treatment may be beneficial. However, at least two factors should be considered when translating these results to clinical practice. First, we used subcutaneous and intravenous routes of administration, which differ from the oral route commonly used in daily buprenorphine treatment (Connery 2015). However, our use of subcutaneous minipumps models the delivery of extended-release buprenorphine formulations, which are administered subcutaneously (Haight et al. 2019). Second, the use of placebos in clinical settings raises both ethical and procedural challenges, including concerns related to insurance coverage, liability, and patient autonomy (Colloca and Howick 2018). These challenges, however, can be addressed through transparent patient-provider communication and the use of open-label placebo protocols supported by informed consent (Colloca et al. 2016).

## Conclusions and future directions

We showed a dose-extending placebo effect in a rat model of opioid maintenance. We observed this effect using two routes of opioid maintenance administration, with remifentanil as the self-administered drug. These findings raise questions for future research. One question is whether this effect generalizes to other commonly misused opioids, such as heroin, oxycodone, and fentanyl. Another question is whether the Cue + Half-dose condition continues to suppress drug self-administration with repeated treatment over days or after intermittent exposure (every few days) during chronic treatment with the Full-dose. It would also be of interest to determine if the dose-extending placebo effect generalizes to chronic treatment with an opioid antagonist such as naltrexone.

Further research should also explore if the dose-extending placebo effect generalize to rat models of methadone maintenance (Chow et al. 2024; Leri et al. 2004) and amphetamine maintenance to reduce cocaine self-administration (Allain et al. 2021). Additionally, dose-extending placebo effects could inform the development of similar dose reduction strategies with non-pharmacological treatments (e.g., rewarding social interaction) initially paired with effective opioid maintenance doses.

From a clinical perspective, our findings suggest that combining placebos with lower medication doses (see Sandler and Bodfish (2008) could mimic the therapeutic effects of higher doses while reducing side effects and overall treatment exposure that go beyond maintenance treatment. This approach may also be relevant for emerging neuromodulation techniques for addiction treatment, which are often influenced by placebo effects (Harel et al. 2022; Perini et al. 2020). Overall, our proof-of-concept study highlights a potential approach for enhancing opioid addiction treatment by leveraging placebo effects to improve efficacy of maintenance medications.

## Data Availability

Raw data and the results of their primary analyses are available on request from Jonathan J. Chow (jonathan.chow@nih.gov) or Yavin Shaham (yavin.shaham@nih.gov).
